# Phytoavailability of Cadmium (Cd) to Pak Choi (*Brassica chinensis* L.) Grown in Chinese Soils: A Model to Evaluate the Impact of Soil Cd Pollution on Potential Dietary Toxicity

**DOI:** 10.1371/journal.pone.0111461

**Published:** 2014-11-11

**Authors:** Muhammad Tariq Rafiq, Rukhsanda Aziz, Xiaoe Yang, Wendan Xiao, Peter J. Stoffella, Aamir Saghir, Muhammad Azam, Tingqiang Li

**Affiliations:** 1 Ministry of Education Key Laboratory of Environmental Remediation and Ecological Health, College of Environmental and Resource Sciences, Zhejiang University, Hangzhou, China; 2 Department of Environmental Science, International Islamic University, Islamabad, Pakistan; 3 Indian River Research and Education Center, Institute of Food and Agricultural Sciences, University of Florida, Fort Pierce, Florida, United States of America; 4 Institute of Statistics, Zhejiang University, Hangzhou, China; 5 College of Agriculture and Biotechnology, Zhejiang University, Hangzhou, China; Chinese Academy of Sciences, China

## Abstract

Food chain contamination by soil cadmium (Cd) through vegetable consumption poses a threat to human health. Therefore, an understanding is needed on the relationship between the phytoavailability of Cd in soils and its uptake in edible tissues of vegetables. The purpose of this study was to establish soil Cd thresholds of representative Chinese soils based on dietary toxicity to humans and develop a model to evaluate the phytoavailability of Cd to Pak choi (*Brassica chinensis* L.) based on soil properties. Mehlich-3 extractable Cd thresholds were more suitable for Stagnic Anthrosols, Calcareous, Ustic Cambosols, Typic Haplustalfs, Udic Ferrisols and Periudic Argosols with values of 0.30, 0.25, 0.18, 0.16, 0.15 and 0.03 mg kg^−1^, respectively, while total Cd is adequate threshold for Mollisols with a value of 0.86 mg kg^−1^. A stepwise regression model indicated that Cd phytoavailability to Pak choi was significantly influenced by soil pH, organic matter, total Zinc and Cd concentrations in soil. Therefore, since Cd accumulation in Pak choi varied with soil characteristics, they should be considered while assessing the environmental quality of soils to ensure the hygienically safe food production.

## Introduction

Cadmium (Cd) is an important environmental pollutant toxic to animals and human beings. It is one of the most mobile elements, among all the toxic heavy metals [1]. Cadmium is not required for plants growth or reproduction, however its bioaccumulation and subsequent accrual in the food chain surpasses all other trace elements due to its high mobility in soil [2]. It is the most toxic element in the environment and even at low concentrations is very toxic to living cells and considered as carcinogenic [3]. In humans, Cd exposure can result in multiple adverse effects, such as testicular damage, renal and hepatic dysfunction, etc. [3]. Moreover, Cd is implicated in the development of cancer, phytotoxic at higher levels of concentrations [4] and classified as a type I carcinogen by the International Agency for Cancer Research [5]. Significant quantities of Cd can be transferred from contaminated soil to plants [6]. Therefore, crops produced from Cd contaminated soils may be unsuitable or even detrimental for animal and human consumption [7].

Vegetables are an important component of human diet since they contain proteins, carbohydrates as well as minerals and vitamins [8]. The proportion of vegetables consumed in the total diet has been increased with the improvement of living standards. However, vegetables are also one of the most important pathways through which heavy metals enter the food chain and affect human health. Leafy vegetables can accumulate higher concentrations of Cd than other crops [9], [10]. Leafy vegetables are known to accumulate higher concentrations of Cd in the edible parts even when grown in soils containing low concentrations of Cd [11]. Pak choi (*Brassica chinensis* L.), also known as Chinese cabbage, is a popular leafy vegetable, grown and consumed worldwide. Therefore, it is imperative to control Cd concentrations in Pak choi, especially in its edible parts to ensure food safety. To limit the transfer of soil Cd into the edible parts of Pak choi, an understanding of its accumulation characteristics is required. Currently, there is an elevated concern over Cd accumulation in food and its potential risks to human health [12]. Cadmium accumulation and distribution varies among vegetable cultivars and tissues [13]. However, the accumulation and distribution of Cd in vegetables grown in a diversity of soil types were rarely studied [14].

About one fifth of agricultural land is contaminated by Cd, lead (Pb) and arsenic (As) in China [15]. Moreover, it was reported that about 20% of farm lands in China are contaminated with heavy metals and Cd contamination accounts for more than 1.3×10^5^ ha of the total affected area [16], [17]. Cadmium uptake by rice (*Oryza sativa* L.) and vegetables from soil is the initial source of exposure for human beings [18], [19]. Therefore, there are environmental concerns of soils, food safety and human health for the present and future agricultural and environmental sustainability of world vegetable supplies. As, only a small fraction of total trace metals in soil is available for plant absorption, it is widely accepted that the total metal content in soils is neither a viable indicator of phytoavailability nor an adequate tool to assess the potential risk of dietary toxicity [12]. Tracy and Sheila [20] reported that extractable Cd content in soil may be an improved indicator of bioavailability and toxicity than the total contents and toxicity and availability of metals differed among soils types. Metal uptake and translocation studies were conducted for different crops under varying soil conditions, to further understanding uptake and the transport mechanisms [21], [22].

To ensure the food safety and environmental quality of soils, guidelines for permissible concentrations of Cd in agricultural soils need to be established. Due to limited number of studies, the soil environmental quality guidelines for heavy metals in farmland soils developed and applied in the world are still based on total metal contents of soil. Minimal attention has been focused on metal accumulation differences among the edible parts of crops, and the relationship between total concentration and phytoavailability of heavy metals in different soil types [4]. Developing the linkage between the bioavailability of Cd in soil and its transfer into the edible plant parts is a key to improving existing soil environmental quality standards. Information is vital on the degree of translocation of heavy metals from soils to plants, which are used as food crops, and absorption of metals in food plants to concentration that does not cause phytotoxicity symptoms [23]. This study was conducted in seven Chinese soil types to establish direct relationship of Cd level in such contaminated soils and Cd uptake in Pak choi. The main objectives were to establish soil Cd thresholds for representative Chinese soils based on human dietary toxicity and to determine the relationships between several soil properties and Cd accumulation in Pak choi. This information will be useful in establishing soil protection guidelines to produce hygienically safe vegetables.

## Materials and Methods

### Ethics Statement

The soils used in this study were agricultural soils. No specific permissions were required for the described locations. We confirm that the field studies did not involve endangered or protected species.

### Soil Collection and Analysis

Seven Chinese soils were selected for this study. Udic Ferrisols, Mollisols, Periudic Argosols, Typic Haplustalfs, Ustic Cambosols, Calcaric Regosols and Stagnic Anthrosols were collected from Chinese cities of Guilin (104°40′–119°45′E, 24°18′– 25°41′N), Harbin (125°42′– 130°10′E, 44°04′–46°40′N), Huzhou (119°14′–120°29′E, 30°22′–31°11′N), Zhanjiang (110°08′–110°77′E, 20°33′–21°62′N), Qufu (116°51′–117°13′E, 35°29′–35°49′N), Ya'an (102°37′–103°12′E, 29°23′–30°37′N) and Jiaxing (120°7′–121°02′E, 30°5′–30°77′N), respectively. Soils samples were taken at a depth of up to 20 cm from the upper horizon. Each sample was air-dried, ground, and screened through two mm sieve before laboratory analysis. Soil pH, cation exchange capacity, organic matter contents, and particle size density were measured by using previously described methods [24]–[27]. Physicochemical properties of these soils are reported (Table 1).

**Table 1:** Basic Chemical and Physical Characteristics of Seven Chinese soils.

Soil Types	Mollisols	Ustic Cambosols	Stagnic Anthrosols	Periudic Argosols	Typic Haplustalfs	Udic Ferrisols	Calcaric Regosols
pH	7.23±0.08	7.80±0.02	6.49±0.03	4.85±0.06	5.16±0.05	4.43±0.07	8.02±0.04
OM (g kg^−1^)	32.2±0.32	7.54±0.20	21.4±0.34	11.6±0.17	6.37±0.56	19.1±0.15	21.8±0.14
CEC (cmol kg^−1^)	34.0±2.51	15.8±1.62	20.2±1.41	12.6±1.52	8.33±2.14	17.3±1.96	25.5±1.46
Total Cd (mg kg^−1^)	0.51±0.02	0.59±0.06	0.79±0.04	0.47±0.02	0.92±0.01	1.06±0.09	0.96±0.06
Total Zn (mg kg^−1^)	31.18±1.47	26.93±0.43	41.36±1.71	15.17±0.88	25.3±1.44	24.6±0.23	28.59±1.38
Sand (%)	20.6±1.54	21.6±1.29	11.4±0.26	24.8±0.65	37.4±0.96	32.75±1.65	31.6±0.57
Silt (%)	60.2±2.21	65.4±2.62	73.0±2.41	58.2±1.04	40.8±1.66	39.8±1.26	44.0±1.26
Clay (%)	19.2±1.24	13.0±1.05	15.6±1.17	17.0±0.34	21.8±0.82	49.6±1.19	24.4±1.32

### Cadmium Spiking and Aging

Soil samples of Mollisols, Periudic Argosols, Stagnic Anthrosols and Ustic Cambosols were spiked with Cd as Cd(NO_3_)_2_ in an aqueous solution at loading rates of 1.0, 2.0, 4.0, 6.0 and 8.0 mg Cd kg^−1^ soil along with an untreated control (Ck), the background values of Cd concentration was below 0.50 mg kg^−1^ in these soil. However, the Udic Ferrisols, Typic Haplustalfs and Calcaric Regosols soil samples, with the background values of Cd concentration above 0.50 mg kg^−1^, were spiked with Cd to establish the contamination levels of 2.0, 4.0, 6.0 and 8.0 mg Cd kg^−1^ soil along with the untreated control (Ck). Soil moisture was maintained up to 70% of its water-holding capacity by using distilled water. All the spiked soils were aged for one year subsequent to greenhouse experimentation. After one year aging period, the concentrations of total Cd, and Mehlich-3 extractable Cd were determined in each of the spiked soils.

### Containerized Experiment

A containerized experiment was performed in greenhouse by growing Pak choi (*Brassica chinensis* L.) during March – April, 2012 at Zhejiang University, Hangzhou, China. Seed of Pak choi was obtained from the Zhejiang Seed Co. Hangzhou, China. Seeds were washed with distilled water and air-dried prior to sowing. Seeds were germinated in dark at 25°C and transplanted into quartz sand bed to establish seedlings. Four healthy, uniform and 21-day-old seedlings were transplanted into plastic containers with a diameter of 18 cm and height of 17 cm. Each container had 3 kg of soil. Fertilizers were applied at the rates of 0.4 g of N as CO (NH_2_)_2_ and 0.2 g P as KH_2_PO_4_ per kg of soil. The experiment was carried out in a completely randomized design (CRD). Treatments were established in triplicate, and the containers were randomly arranged in a greenhouse bench under controlled conditions of 16 h of light at 30°C and 8 h of dark at 22°C. Plants were monitored daily and watered as necessary.

### Plant Sample Collection

Pak choi was harvested after 30 days from transplanting. The plants of Pak choi were removed from each container and separated into root and shoots (including stems and leaves). Roots and shoots of Pak choi were first washed with tap water and then with ultrapure distilled water, to remove all visible soil particles. Clean plant samples were first blotted dry, and then dried at 70°C for 72 h in an oven. Dry shoot weight of samples was recorded. Dry plant samples were ground to pass through a 60 mm sieve using an agate mill prior to Cd concentration analysis.

### Total Cd of Soil and Plant

For determination of total Cd concentration in soil, 0.20 g of soil samples was digested with HNO_3_–HF –HClO_4_ (5∶1∶1) [4]. For plant samples, 0.20 g of shoots for each treatment was digested with HNO_3_–H_2_O_2_ (5∶1). After cooling the digest was transferred to a volumetric flask, diluted with distilled water to 50 mL [28]. The concentrations of Cd in the filtrate were determined using inductively coupled plasma*–*mass spectrometry (ICP-MS, Agilent, 7500a, CA, USA). The ICP-MS was operated at the following conditions: the radio frequency power at the torch 1.2 kW, the plasma gas flow 15 L min^−1^, the auxiliary gas flow 0.89 L min^−1^, and the carrier gas flow 0.95 L min^−1^ [28]. The same procedure without samples was used as control and three replications were conducted for each sample.

### Mehlich-3 Extractable Cd in Soils

Mehlich-3 extractable Cd in soils was determined following the extraction method described by Mehlich [29]. Briefly, 5 g (0.2 mm sieved) of dry soil was shaken with 50 mL of Mehlich-3 solution (0.2 mol L^−1^ CH_3_COOH, 0.25 mol L^−1^ NH_4_NO_3_, 0.015 mol L^−1^ NH_4_F, 0.013 mol L^−1^ HNO_3_, 0.001 mol L^−1^ EDTA) for 5 min (200 rpm) at 25°C. The suspension was centrifuged at 5000 rpm for 10 min and filtered through 0.45 µm filter paper. The same procedure without samples was used as control and three replications were conducted for each soil sample. The Cd concentration in the filtrate was analyzed by inductively coupled plasma*–*mass spectrometry (ICP-MS, Agilent 7500a, CA, USA).

### Quality Control for Cd Analysis

Quality assurance and quality control (QA/QC) for Cd in soil and Pak choi were conducted by determining Cd contents in the certified reference materials (soil GSBZ 50013-88 and plant GBW- 07402) respectively, approved by General Administration of Quality Supervision, Inspection and Quarantine of the People's Republic of China (AQSIQ) and National Center for Reference Materials. The analytical results showed a recovery rate of 97.3% and 102.1% respectively.

### Derivation of Soil Cd Thresholds for Potential Dietary Toxicity in Pak choi

For ensuring the environmental and food safety for human beings, an effort has been made to develop guidelines for acceptable concentrations of potentially harmful Cd in seven agricultural soils types of China. In this context, the amounts of Cd in Pak choi above than threshold level of food safety are adversely affecting humans are critical. Since, Cd bioavailability differed among soil types, the focus was on the development of soil Cd thresholds for representative Chinese soils based on food safety, Provisional Tolerable Weekly Intake (PTWI) of Cd recommended by FAO/WHO Joint Expert Committee on Food Additives, is 7 µg kg^−1^ of body weight [30]. Estimated daily intake of metal (EDIM) was determined by the following equation. 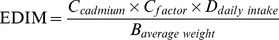
Where, *Cc_admium_, C_factor,_ D_food intake_ and B_average weight_* represent average Cd concentration in Pak choi (mg kg^−1^), conversion factor, daily consumption of Pak choi (g) and average body weight (kg) of the adult consumers, respectively. Average daily consumption of Pak choi for adults was considered to be 0.345 kg person^−1^ d^−1^ [31] and a conversion factor 0.085 was used to convert fresh Pak choi weight to dry weight [32]. Average body weight of adult was considered to be 60 kg as motioned in previous reports [30]. According to the above equation of EDIM, the provisional tolerable daily tolerable intake of Cd for Pak choi was 2.04 mg kg^−1^ on a dry weight basis. Soil Cd threshold levels for potential dietary toxicity from Pak choi were calculated according to the tolerable daily dietary intake level of Cd (2.04 mg kg^−1^) and the regression equations.

### Statistical Analysis

Stepwise multiple regression analysis, single linear regression and one-way analysis of variance (ANOVA) were performed using the statistical software package SPSS (version 18.0). All values reported in this work are means of three independent replications. Treatment means were separated by least significant difference (LSD) test, at 5% level.

## Results

### Characteristics of Soils

Soils evaluated were representative of most of Chinese soil types, pH range of soils were strongly acidic to mild alkaline. Chemical and physical characteristics varied among the seven soils. Highest total Cd and Zn concentrations (background value) were observed in Udic Ferrisols and Stagnic Anthrosols respectively. Mollisols contained the highest amount of organic matter and exhibited an elevated cation exchange capacity as well (Table 1).

### Mehlich-3 Extractable Cd in Soils after Aging of 1 Year

Mehlich-3 extractable Cd content increased significantly with increasing Cd spiking levels in all the seven soils. Mehlich-3 extractable Cd ranged from 0.16–3.95 mg kg^−1^ in these soils under different Cd levels (Table 2). The Cd contents varied significantly among these soils, decreasing in order: Periudic Argosols> Typic Haplustalfs> Udic Ferrisols> Stagnic Anthrosols> Mollisols> Ustic Cambosols> Calcaric Regosols. Mehlich-3 extractable Cd concentration was greater at higher rates of Cd spiking in each soil. These results indicated that minimum and maximum extractability of Cd was found in Calcaric Regosols and Periudic Argosols, respectively under the highest (8 mg kg^−1^) level of Cd spiked. Mehlich-3 extractable concentrations were dependent on total Cd in each soil, however the extractability was significantly higher in low pH soils as compared to the medium and high pH soils (Table 2).

**Table 2:** Mehlich-3 Extractable Cd Contents (mg kg^−1^) in Seven Chinese Soils at the onset of Containerized Experiment after Aging of 1 year.

Cd conc. (mg kg^−1^)	Mollisols	Ustic Cambosols	Stagnic Anthrosols	Periudic Argosols	Typic Haplustalfs	Udic Ferrisols	Calcaric Regosols
Ck	0.19±0.05c	0.17±0.07c	0.22±0.08d	0.16±0.06e	0.31±0.12d	0.42±0.09d	0.29±0.07c
1	0.21±0.04c	0.19±0.06c	0.30± 0.04d	0.41±0.13e	-	-	-
2	0.62±0.04bc	0.48±0.10c	0.56±0.27d	0.99±0.29d	0.69±0.17d	0.65±0.11d	0.35±0.06c
4	1.19±0.08b	1.05±0.90bc	1.17±0.24c	1.79±0.54c	1.52±0.26c	1.46±0.60c	0.71±0.10bc
6	2.21±0.91a	2.13±0.90b	2.01±0.47b	2.98±0.46b	2.44±0.64b	2.39±0.90b	1.38±0.29ab
8	2.89±1.04a	3.28±0.84a	3.29±0.73a	3.95±0.41a	3.58±0.53a	3.47 ±0.70a	2.01±0.94a

Mean values followed by different letters within the same column are significantly different at *P* <0.05.

### Biomass Yield of Pak choi

Generally, Pak choi had tolerance to Cd toxicity in Mollisols, Stagnic Anthrosols and Calcaric Regosols soils, indicating low phytoavailability of Cd in these soils. Shoot biomass of Pak choi under different Cd treatments of these soils did not decrease significantly as compared with their respective controls. However, the shoot biomass of Pak choi grown in Ustic Cambosols, Udic Ferrisols, Periudic Argosols and Typic Haplustalfs decreased significantly as compared with the control indicating higher phytoavailability of Cd in these soils (Table 3). The stimulating effect of Cd on shoot biomass of Pak choi occurred at 1 mg kg^−1^ and 2 mg kg^−1^ in Ustic Cambosols and Mollisols respectively, whereas in Stagnic Anthrosols, it occurred at 4 mg kg^−1^. The dry weight of Pak choi shoots at 8 mg kg^−1^ Cd generally decreased in order of: Calcaric Regosols> Mollisols, Stagnic Anthrosols> Ustic Cambosols> Udic Ferrisols> Periudic Argosols> Typic Haplustalfs (Table 3).

**Table 3:** Dry Biomass (g plant^−1^) of Pak choi Shoots Grown on Seven Chinese Soils with Different Loading Rates of Cd.

Cd conc. (mg kg^−1^)	Mollisols	Ustic Cambosols	Stagnic Anthrosols	Periudic Argosols	Typic Haplustalfs	Udic Ferrisols	Calcaric Regosols
Ck	1.95±0.18a	1.54±0.13a	1.13±0.31a	0.84±0.29a	0.73±0.20a	0.95±0.11a	2.07±0.46a
1	1.91±0.37a	1.66±0.59a	1.10±0.30a	0.63±0.35ab	-	-	-
2	2.22±0.58a	1.02±0.49a	1.14±0.53a	0.59±0.47ab	0.54±0.41ab	0.79±0.23ab	1.98±0.11a
4	1.89±0.49a	1.47±0.08a	1.25±0.28a	0.51±0.03ab	0.43±0.38ab	0.58±0.06abc	1.95±0.06a
6	1.86±0.48a	1.35±0.13ab	1.09±0.09a	0.36±0.02ab	0.38±0.11ab	0.50±0.24bc	1.90±0.15a
8	1.54±0.28a	0.95±0.15b	1.11±0.51a	0.17±0.06b	0.10±0.01b	0.39±0.29c	1.75±0.17a

Mean values followed by different letters within the same column are significantly different at *P* <0.05.

### Accumulation and Distribution of Cadmium in Pak choi

Cadmium concentration in the shoots and roots of Pak choi varied significantly among soils at different Cd levels and soil types. Roots exhibited the higher Cd contents as compared with Pak choi shoots. The content of Cd enhanced with increasing Cd loading rate in the soils. Cd concentration was high in the roots (2.42 to 169. 95 mg kg^−1^ DW), while low in the shoot (0.48 to 89.21 mg kg^−1^ DW) (Table 4). Cd uptake in Pak choi tissues was affected by soil type, primarily due to the variation in Cd bioavailability. The lowest and highest Cd concentrations in the Pak choi tissues were at the highest (8 mg kg^−1^) level of Cd in Calcaric Regosols and Periudic Argosols, respectively. Cadmium concentrations in Pak choi followed an order of: Periudic Argosols> Typic Haplustalfs> Udic Ferrisols> Ustic Cambosols> Mollisols> Stagnic Anthrosols> Calcaric Regosols at 8 mg kg^−1^ soil Cd level (Table 4).

**Table 4:** Cd Concentration (mg kg^−1^ DW) in Pak choi Grown under Different Cd Levels in Seven Chinese Soils.

Cd (mg kg^−1^)	Mollisols		Ustic Cambosols		Stagnic Anthrosols		Periudic Argosols		Typic Haplustalfs		Udic Ferrisols		Calcaric Regosols	
	Root	Shoot	Root	Shoot	Root	Shoot	Root	Shoot	Root	Shoot	Root	Shoot	Root	Shoot
Ck	3.41±0.64e	1.00±0.28e	2.42±0.45f	0.48±0.22e	2.73±0.28e	0.85±0.12e	7.26±1.10f	1.84±0.56f	12.35±1.68e	4.37±1.02e	11.81±0.78e	6.77±0.92e	3.11±0.66e	1.92±0.58b
1	5.09±0.91e	2.53±0.76e	6.69±0.65e	2.06±0.98e	4.12±0.75e	1.92±0.94e	21.3±2.01e	11.00±1.57e	-	-	-	-	-	-
2	8.92±1.09d	4.31±0.54d	9.30±1.29d	4.77±0.22d	8.34±1.11d	3.94±0.83d	39.33±3.86d	28.51±2.61d	25.41±1.79d	12.09±1.34d	19.41±1.27d	10.06±1.10d	4.24±0.99d	2.70±0.99b
4	14.21±0.90c	8.34±1.11c	14.00±1.43c	9.01±1.06c	13.21±1.55c	7.06±1.06c	68.43±3.71c	39.12±3.06c	39.11±2.28c	32.51±2.51c	33.55±1.71c	20.89±1.46c	10.37±1.21c	3.46±1.10b
6	21.24±1.20b	11.75±1.17b	24.36±1.11b	17.23±1.50b	20.21±1.80b	11.63±1.54b	117.76±6.53b	68.21±4.08b	68.00±3.96b	41.11±3.01b	55.53±2.67b	31.45±2.48b	14.41±1.03b	6.57±0.93a
8	38.96±1.39a	18.11±1.12a	43.21±2.21a	22.22±1.51a	28.56±1.42a	17.63±1.37a	169.95±8.11a	89.21±5.70a	118.21±6.24a	69.21±4.11a	85.32±4.28a	53.73±2.88a	18.73±1.54a	8.13±0.87a

Mean values followed by different letters within the same column are significantly different at *P* <0.05.

### Relationship between Mehlich-3 Extractable Cd in Soils and Pak choi Cd Content

Cadmium concentrations in shoots of Pak choi were significantly correlated to total Cd and Mehlich-3 extractable Cd contents in soils (*R^2^* = 0.95 to 0. 99, and 0.97 to 0.99 respectively). Cadmium concentrations in Pak choi shoots were best related to total Cd content in Mollisols (*R^2^* = 0.99). Whereas, the Cd concentrations of Pak choi shoots were best correlated to Mehlich-3 extractable Cd in Ustic Cambosols, Stagnic Anthrosols Periudic Argosols, Udic Ferrisols, Typic Haplustalfs and Calcaric Regosols with *R^2^* = 0.97, 0.99, 0.99,0.98,0.99 and 0.98, respectively (Table 5).

**Table 5:** Regression Correlation between Cd Contents in the Edible Shoots of Pak choi and Different Forms of Cd in Various Soils.

Soil type	Form of Soil Cd	Regression equation	*R^2^*
Mollisols	Total Cd	y = 2.1706x +0.1669	0.99
	Mehlich-3 extractable Cd	y = 5.7207x +0.7037	0.98
Ustic Cambosols	Total Cd	y = 2.9358x −0.9586	0.96
	Mehlich-3 extractable Cd	y = 7.0409x +0.7648	0.97
Stagnic Anthrosols	Total Cd	y = 2.2344x −0.5651	0.98
	Mehlich-3 extractable Cd	y = 5.3848x +0.4458	0.99
Periudic Argosols	Total Cd	y = 11.061x +0.6961	0.98
	Mehlich-3 extractable Cd	y = 22.326x +1.3968	0.99
Typic Haplustalfs	Total Cd	y = 8.7318x −4.0825	0.97
	Mehlich-3 extractable Cd	y = 19.123x −0.8046	0.98
Udic Ferrisols	Total Cd	y = 6.6697x −2.7793	0.97
	Mehlich-3 extractable Cd	y = 14.873x −0.3773	0.99
Calcaric Regosols	Total Cd	y = 0.8936x +0.924	0.95
	Mehlich-3 extractable Cd	y = 3.5937x +1.1512	0.98

### Soil Cd Thresholds for Potential Dietary Toxicity in Pak choi

Total soil Cd thresholds for potential dietary toxicity from the consumption of Pak choi conformed to an order of: Calcaric Regosols> Stagnic Anthrosols> Ustic Cambosols> Mollisols> Udic Ferrisols> Typic Haplustalfs> Periudic Argosols, and were 1.25, 1.16, 1.02, 0.86, 0.72, 0.70 and 0.12 mg kg^−1^, respectively. Mehlich-3 extractable Cd thresholds were 0.30, 0.25, 0.23, 0.18, 0.16, 0.15 and 0.03 mg kg^−1^ and decreased in the following order of: Stagnic Anthrosols> Calcareous> Mollisols> Ustic Cambosols> Typic Haplustalfs> Udic Ferrisols>Periudic Argosols, respectively (Table 6).

**Table 6:** Soil Cd Threshold Levels for Potential Dietary Toxicity in Edible Part of Pak choi Calculated from the Permissible Limit of Cd (2.04 mg kg^−1^ DW) in Leafy Vegetables and Regression Equations.

Soil Type	Total Cd (mg kg^−1^)	Mehlich-3 extractable Cd (mg kg^−1^)
Mollisols	0.86	0.23
Ustic Cambosols	1.02	0.18
Stagnic Anthrosols	1.16	0.30
Periudic Argosols	0.12	0.03
Udic Ferrisols	0.72	0.16
Typic Haplustalfs	0.70	0.15
Calcaric Regosols	1.25	0.25

## Discussion

### Biomass Yield of Pak choi

Dry weight of Pak choi did not decrease significantly under different Cd levels (Ck to 8.0 mg kg^−1^) in Mollisols, Stagnic Anthrosols and Calcaric Regosols and even increased at 1, 2 and 4.0 mg kg^−1^of treatment levels. Similar stimulatory responses of biomass to Cd exposure have also been reported in several plant species [33], [34]. The stimulatory effect of Cd on plant biomass may be explained by various mechanisms, for examples, metal ions can serve as enzyme activators in cytokinins metabolism, which stimulates the growth of plants, [35] and a low dose of metal exposure may cause changes in cytokinins and plant hormones that regulate growth and development of plants [36]. Kaminek [36] reported that cytokinins may delay senescence by maintaining chlorophyll production and photosynthetic activity in plant leaves.

Cd exposure may cause changes to various physiological and biochemical processes in plant tissues, such as, reduction in dry biomass may be due to the negative effects of Cd on the roots, and plants could not take up nutrients to continue their normal activities. It has been well reported that Cd can reduce plant growth and development by interfering in various metabolic processes, such as, inhibition of the proton pump, reduction in root elongation, and damage to photosynthetic activity [37], [38]. The excess amount of Cd in soil may be responsible for causing disturbances in mineral nutrition and carbohydrate metabolism [39].

Shoot biomass of Pak choi grown in Ustic Cambosols, Udic Ferrisols, Periudic Argosols and Typic Haplustalfs decreased significantly as compared with the control. The inhibitory effect of Cd on shoot growth is consistent with earlier reports of three Chinese cabbage cultivars exposed to different soil Cd levels of 1, 2.5 and 5 mg kg^−1^. A significant decrease in the shoot biomass was observed at 2.5 and 5 mg kg^−1^ levels of Cd as compared to their respective controls [40]. Shentu *et al*. [4] found a 46% reduction in root dry weight of radish at 6.31 mg kg^−1^ Cd exposure in red yellow soil, which is in accordance with our results as we also noticed a shoot dry weight reduction of 58.9%, 79.7%, and 86.3% in Udic Ferrisols, Periudic Argosols and Typic Haplustalfs respectively at 8 mg kg^−1^ level of soil Cd as compared to their respective controls.

### Accumulation and Distribution of Cadmium in Pak choi

Variations of Cd accumulation in Pak choi grown in different soils with different pH may be due to the difference in bioavailability of Cd in each soil. Liang *et al*. [13] stated that Cd content of spinach plants was highly dependent upon the soil pH being highest at pH 5.3. Lai and Chen [41] reported that Cd concentration in Pak choi shoots was up to 85 mg kg^−1^ DW with an application of soil Cd up to 20 mg kg^−1^. Moreover, it was observed that accumulation of the Cd in rice shoot ranged from 67.9 to 241.7 µg/pot in different rice genotypes at 5 mg kg^−1^ soil Cd level [42].

### Relationship between Mehlich-3 Extractable Cd in Soils and Pak choi Cd Content

Mehlich-3 extraction technique appeared efficient to assess Cd phytoavailability to Pak choi, grown in seven textured soils, as evidenced by high correlation coefficients (*R^2^*>0.97). This is in agreement with our previous studies, [43], [28] which reflected a high linear correlation (*R^2^*>0.98) between Mehlich-3 Cr and Cr contents in Pak choi and rice grown under six different textured soils. These results are similar to those reported in which extractable soil metal was an improved indicator for Cd phytoavailability in several vegetable crops [4]. Mehlich-3 extraction method is applicable to a large range of soil types, from acidic to alkaline, and makes it ideal for application at a wide scale [44]. Generally, the extraction techniques are assumed to have a relationship between the extractable fraction of metals and the phytoavailability of the metals to plants, and these metals such as exchangeable, soluble, and loosely adsorbed metals are labile and thus readily available to plants [12], [45]. The efficiency of Mehlich-3 extraction method was compared with the EPA 3050 B method (a strong acid digestion method) to assess the predictive capabilities through a lettuce (green specie) bioassay. Mehlich-3 extraction was positively correlated with the more costly EPA test, and could be developed as a less expensive and easily conduct able technique [46].

### Soil Cd Thresholds for Potential Dietary Toxicity in Pak choi

Cadmium concentrations in the shoots of Pak choi were significantly correlated to total Cd and Mehlich-3 extractable Cd contents in soils, with *R^2^* values of 0.95 to 0.99, and 0.97 to 0.99, respectively. From this investigation, Cd contents in Pak choi shoots were correlated to total Cd content in Mollisols (*R^2^* values of 0.99). Cadmium concentrations of Pak choi shoots were highly correlated to Mehlich-3 extractable Cd in Ustic Cambosols, Stagnic Anthrosols, Periudic Argosols, Udic Ferrisols, Typic Haplustalfs and Calcaric Regosols with *R^2^* values of 0.97, 0.99, 0.99, 0.98, 0.99 and 0.98, respectively. Total Cd threshold levels for potential dietary toxicity conformed to an order of: Calcaric Regosols> Stagnic Anthrosols> Mollisols> Ustic Cambosols> Typic Haplustalfs> Udic Ferrisols> Periudic Argosols and were 1.25, 1.16, 1.02, 0.86, 0.72, 0.70 and 0.12 mg kg^−1^, respectively. Mehlich-3 extractable Cd thresholds decreased in the following order of: Stagnic Anthrosols> Calcareous> Mollisols> Ustic Cambosols> Udic Ferrisols>Typic Haplustalfs> Periudic Argosols and were 0.30, 0.25, 0.23, 0.18, 0.16, 0.15 and 0.03 mg kg^−1^, respectively (Table 6).

Cadmium concentrations in Pak choi shoots, were highly correlated to total Cd content in Mollisols with the threshold levels of 0.86 mg kg^−1^ with a *R^2^* = 0.99. However, the Cd concentrations of Pak choi shoots were best related to Mehlich-3 extractable Cd in Stagnic Anthrosols, Calcaric Regosols, Mollisols, Ustic Cambosols, Typic Haplustalfs and Periudic Argosols with thresholds values of 0.30, 0.25, 0.23, 0.18, 0.16, 0.15 and 0.03 mg kg^−1^, (*R^2^* values of 0.97, 0.99, 0.99, 0.98, 0.98 and 0.98), respectively. Based on the wide range of applicability and the simplicity of extraction method, it is proposed that Mehlich-3 extractable Cd is more suitable to be used as soil Cd thresholds for potential dietary toxicity in Pak choi. Our previous study evaluated the phytoavailability of Cd to rice, and demonstrated the suitability of Mehlich-3 extraction method in different textured soils [47]. Similar to our results, Murakami *et al*. [48] reported that Mehlich-3 extractable Cd was an improved indicator than total soil Cd and HCl-extractable Cd to predict the grain Cd content of japonica rice varieties. Our results are also in agreement with Shentu *et al*. [4] who also concluded that extractable Cd was a better soil test index for Cd phytoavailability of several vegetables and could be used as soil Cd thresholds for food safety. Among the predicted thresholds (total soil Cd) the lowest value (0.12 mg kg^−1^) was observed for the Periudic Argosols, an acidic soil. Bioavailability and uptake of Cd are very high in this soil. The leafy vegetables like Pak choi can accumulate large quantities of Cd as compared to other crops [9], [10]. Therefore the predicted threshold is even lower than background value of Cd in soil; it means that there is a risk for dietary toxicity from Pak choi grown on it, even with the background value of total Cd in soil. This kind of information has been reported in our previous study. The threshold of total soil Cd for rice was 0.21 mg kg^−1^ which was also lower than background value of total Cd in soil [47].

Cd levels (Ck, 1, 2, 4, 6, 8 mg kg^−1^) used in this investigation represented uncontaminated, lightly contaminated, and moderately Cd polluted soils. Therefore, these levels of Cd contamination are realistic, comparable to those applied in other soil safety risk assessment studies, and thus, the results are applicable in field conditions as well.

### Stepwise Regression Model for Predicting Cd Phytoavailability to Pak choi

Many physicochemical properties of soils can influence the heavy metal accumulation in vegetables. For example, the amount of heavy metal uptake from soils was influenced by soil pH, organic matter (OM) content, cation exchange capacity (CEC) and soil texture [49]. The combinations of basic soil properties may explain Cd uptake by plants [50]. By considering this aspect, soil pH, OM, CEC, total soil Cd, total Zn and clay contents were integrated to simulate the combined effects of soil environment on Cd phytoavailability to Pak choi. Stepwise linear regression was conducted and four independent variables pH, total Zn, OM and total Cd significantly influenced the accumulation of Cd in Pak choi plants (Table 7). Both the multiple correlation and partial regression coefficients reached the statistically significant levels at least the 0.05. For multiple linear regression analyses, *R^2^* values could be used to explain variation of the dependents [12]. It was found that *R^2^* value was above 0.97, which means that more than 97% of variation in Cd concentration in Pak choi shoots could be attributed to soil pH, total Zinc, OM and total Cd contents in soils (Table 7).

**Table 7:** Stepwise Regression Model for Predicting Cd Concentration (Y) in Edible Part of Pak choi based on Soil Properties.

Stepwise regression model	*R^2^*	*F value*	*T value and R^2^ of partial regression coefficient*		
				*T value*	*R^2^*
Y = −39.256–15.516 pH−0.944 Zn_T_−0.379 OM +26.752 Cd_T_	0.977	138.808*	pH	−18.682**	0.964
			Zn_T_	−3.788**	0.524
			OM	−3.652*	0.376
			Cd_T_	2.67*	0.354

a Cd_T_ and Zn_T_ refer to the total Cadmium and Zinc concentrations.

b Superscripts * and ** indicate significant levels of probability at 0.05 and 0.01, respectively.

The influence of each factor on Cd concentration of Pak choi (Y) shoots could be further explained by the values of each coefficient [12]. Stepwise regression model revealed that Cd concentration in the Pak choi was enhanced by lower soil pH (negative coefficients showed negative effect and vice versa), total Zinc, OM contents and higher total soil Cd. Lower soil pH, zinc, OM and higher soil total Cd are among the factors which enhance the bioavailability Cd contents in soils. Therefore, these three variables had the contradictory effect on Cd phytoavailability to Pak choi. Wang *et al.* [12] reported that soil characteristics (e.g. pH, CEC and OM) affected the phytoavailability of different heavy metals in soils, and such influences could be considered in the assessment of phytoavailability of heavy metals. There are four parameters involved in this model and then interactions between them were obvious (e.g. Cd concentration in the extractable fraction was correlated with lower soil pH, soil zinc and OM content). Furthermore, the coefficients obtained in the present model can regulate these cross effects and result in an improved model fitting. For example, there was a negative correlation between the soil pH, Zinc and OM, these factors had an inverse effect on Cd phytoavailability and soil Cd was the leading factor influencing Cd phytoavailability to Pak choi (coefficient of soil Cd was greater than those of pH, Zinc and OM). Our results about soil Cd and pH are in accordance with our recent study which developed an empirical model to correlate the Cd phytoavailability to rice with several soil properties. Soil pH and bioavailable soil Cd were major influencing factors which (pH negatively and soil Cd positively) correlate with the Cd phytoavailability, however total Zn and OM were not included in our previously developed model [47]. Eriksson and Soderstrom [51] reported that the Cd concentration of wheat grain grown on non-calcareous soils of Sweden was positively correlated to soil total Cd and negatively to extractable Zn. A study was conducted on Cd contaminated soils in Taiwan, whereas regression equation was developed to predict Cd concentrations in rice roots by available fractions of Cd and Zn in soil [52]. The negative coefficient of Zn indicated that soil Zn suppressed the uptake of Cd by rice roots in all varieties as Zn has an antagonistic effect on Cd uptake by root [53]. Oliver *et al*. [54] also observed a significant decrease of Cd up to 50% in wheat grain when 2.5–5.0 kg Zn ha^−1^ was applied to Cd contaminated Australian soils.

Organic matter content was negatively correlated with the accumulation of Cd in Pak choi shoots (Table 7). Organic matter plays an important role in determining the bioavailability and mobility of heavy metals in soils. Organic matter is involved in supplying organic chemicals to the soil solution, which may act as chelates and increase metal bioavailability to plants [55]. However, OM could reduce the bioavailability of heavy metals in soils by adsorption or forming stable complexes with humic substances [56]. Halim *et al*. [57] reported that addition of humic acid demonstrated a decrease in extractable heavy metal fraction in metal contaminated soils. This could partially explain the negative correlation of organic matter contents and Cd uptake observed in our present study.

## Conclusions

The present study concludes that Cd concentration in Pak choi tissues was dependent on soil type. To establish the soil Cd thresholds of potential dietary toxicity from Pak choi, both Cd bioavailability in garden soils and Pak choi tissues should be taken into consideration. The selection of proper soil types for vegetable production can help us to avoid the toxicity of Cd in our daily diet. Stepwise regression model demonstrated that soil pH, organic matter, total Cd and Zinc contents may be the major factors having influence on the phytoavailability of Cd in different textured soils.
